# Supplementary material for: The STING-IFN-β-Dependent Axis Is Markedly Low in Patients with Relapsing-Remitting Multiple Sclerosis
Source: Int J Mol Sci. 2020 Dec 4;21(23):9249. doi: 10.3390/ijms21239249 (PMC7730283; doi:10.3390/ijms21239249)
Supplement: Supplementary file 1 [file ijms-21-09249-s001.pdf]

International Journal of
Molecular Sciences

Supplementary materials

The STING-IFN-ß-dependent axis is markedly low in patients with relapsing-remitting multiple sclerosis

Lars Masanneck [1,§], Susann Eichler [1,§,*], Anna Vogelsang [1], Melanie Korsen [2], Heinz Wiendl [1], Thomas Budde [3] and Sven G. Meuth [1,2]

[1] Department of Neurology with Institute of Translational Neurology, University Hospital Münster, 48149 Münster, Germany; lars.masanneck@uni-muenster.de (L.M.); susann.eichler@ukmuenster.de (S.E.); anna.vogelsang@ukmuenster.de (A.V.); heinz.wiendl@ukmuenster.de (H.W.)
[2] Department of Neurology, Heinrich Heine University Düsseldorf, 40225 Düsseldorf, Germany; melanie.korsen@med.uni-duesseldorf.de (M.K.); meuth@uni-duesseldorf.de (S.G.M.)
[3] Institute of Physiology I, University of Münster, 48149 Münster, Germany; tbudde@uni-muenster.de (T.B.)
§ These authors contributed equally to this work.

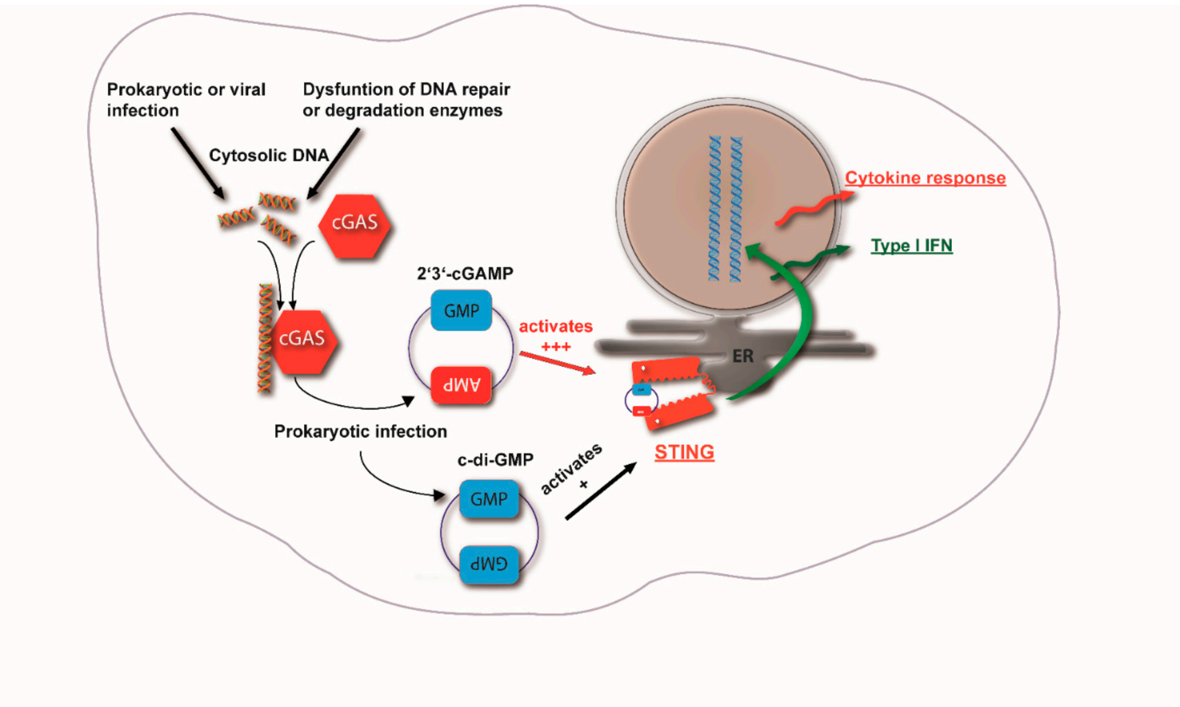

Figure S1. Graphical synopsis of the general mechanism of the STING/IFN-β-axis. The recognition of cytosolic DNA, which can derive from viral or bacterial infections and/or from the lack or dysfunction of DNA repair and degradation enzymes, indicates a pathological state for the affected cell. A vital mechanism for sensing cytosolic DNA involves cyclic GMP-AMP synthase (cGAS), which can produce 2′3′-cGAMP, an endogenous CDN, upon DNA binding. Most commonly, CDN, such as c-di-GMP, are found as signaling molecules in the metabolism of prokaryotes and indicate an infection when present in the human cytosol. With an even higher affinity than its bacterial counterparts, the mammalian second messenger CDN 2′3′-cGAMP binds and activates the endoplasmic reticulum-resident sensor STING. Once STING is activated by binding a CDN in between its dimers, it recruits TANK-binding kinase 1 (TBK1), and a translocation and signaling cascade are initiated. Among other effects, TBK1 phosphorylates interferon regulatory factor 3 (IRF3), which in turn coordinates with nuclear factor 'kappa-light-chain-enhancer' of activated B-cells (NF-κB) to orchestrate an IFN type I response. The IFN response is supported by the excretion of other cytokines. In summary, the cGAS-STING axis is indispensable for detecting pathological DNA, leading to a subsequent orchestration of an anti-infective response to these potentially pathological signals.

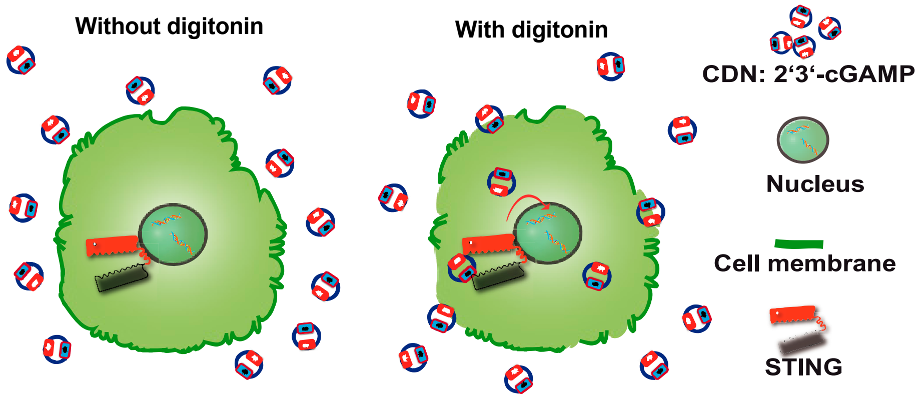

Figure S2. Principle of digitonin permeabilization. CDN, which are used to activate the endoplasmic reticulum resident STING, are hydrophilic molecules that are unlikely to passively cross the plasma membrane. Due to its characteristics as a non-ionic detergent, the glycoside digitonin interacts with the cholesterol of mammalian cell membranes, resulting in a temporary permeabilization of cholesterol-containing membranes. Digitonin permeabilization enables the CDN to enter the cell through a porous plasma membrane and reach their target STING.

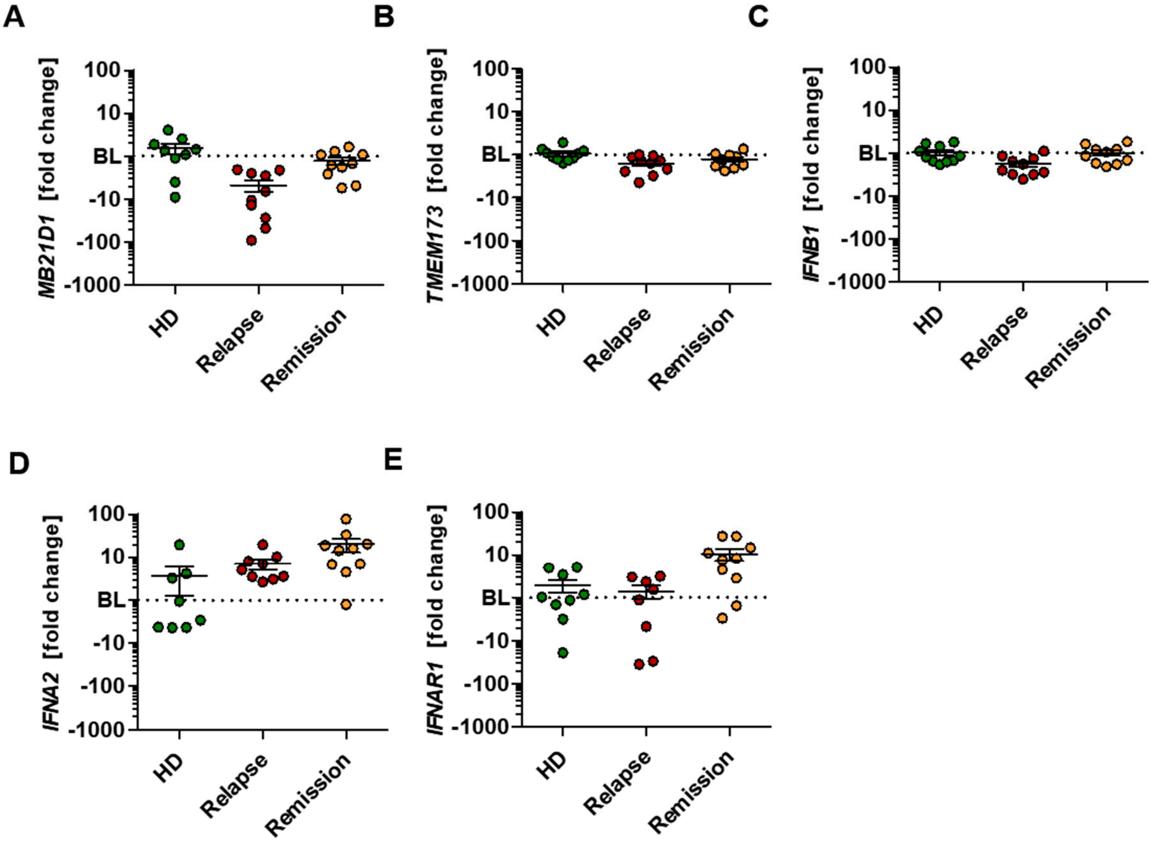

Figure S3. Gene expression changes of STING/IFN-β-axis in RRMS patient-derived PBMC expressed as fold changes. (**A-E**) The gene expression levels of *MB21D1* (**A**), *TMEM173* (**B**), *IFNB1* (**C**), *IFNA2* (**D**), and *INFAR1* (**E**) were analyzed in PBMC of HD and relapsing-remitting multiple sclerosis patients in relapse or remission. Gene expressions are illustrated as fold changes calculated in reference to the HD group. Each data point represents an individual HD or patient.
